# Following 411 Cochrane Protocols to Completion: A Retrospective Cohort Study

**DOI:** 10.1371/journal.pone.0003684

**Published:** 2008-11-10

**Authors:** Andrea C. Tricco, Jamie Brehaut, Maggie H. Chen, David Moher

**Affiliations:** 1 Chalmers Research Group, Children's Hospital of Eastern Ontario Research Institute, Ottawa, Ontario, Canada; 2 Institute of Population Health, University of Ottawa, Ottawa, Ontario, Canada; 3 Department of Epidemiology and Community Medicine, Faculty of Medicine, University of Ottawa, Ottawa, Ontario, Canada; 4 Clinical Epidemiology Program, Ottawa Health Research Institute, Ottawa, Ontario, Canada; 5 Department of Biostatistics, University of Toronto, Toronto, Ontario, Canada; Assiut University Hospital, Egypt

## Abstract

**Background:**

Cochrane reviews are regarded as being scientifically rigorous and are increasingly used by a variety of stakeholders. However, factors predicting the publication of Cochrane reviews have never been reported. This is important because if a higher proportion of Cochrane protocols with certain characteristics (e.g., funding) are being published, this may lead to inaccurate decisions. We examined the frequency of published and unpublished Cochrane reviews and protocol factors that predict the publication of Cochrane reviews.

**Methodology/Principal Findings:**

Retrospective cohort study of Cochrane protocols published in 2000 (Issues 2 to 4) and 2001 (Issue 1). The publication status of these reviews was followed up to Issue 1, 2008 in The Cochrane Library. Survival analysis of the time from protocol publication to the first review publication and protocol factors predicting the time to publication was conducted. There were 411 new Cochrane protocols in the cohort. After excluding 39; 71/372 (19.1%) were unpublished and 301/372 (80.9%) were published as full Cochrane reviews at the time of study analysis (January 2008). The median time to publication was 2.4 years (range: 0.15 to 8.96). Multivariate analyses revealed that shorter time to publication was associated with the review subsequently being updated (hazard ratio, HR: 1.80 [95% confidence interval, CI: 1.39 to 2.33 years]) and longer time to publication was associated with the review having two published protocols, indicating changes to the review plan (HR: 0.33 [95% CI: 0.12 to 0.90 years]).

**Conclusions/Significance:**

Only about 80% Cochrane protocols were published as full reviews after over 8 years of follow-up. The median time to publication was 2.4 years and some reviews took much longer. Strategies to decrease time to publication should be considered, such as streamlining the review process, increased support for authors when protocol amendments occur, and better infrastructure for updating Cochrane reviews.

## Introduction

The mission of the Cochrane Collaboration is to conduct systematic reviews in all areas of healthcare [1]. Currently, the Collaboration includes more than 10,000 members globally organized into clinical review groups (CRGs; e.g., schizophrenia group), methods groups (e.g., bias methods group), and fields (e.g., child health field) [1]. Evidence suggests that Cochrane reviews are the most scientifically reported systematic reviews [2]. They are also increasingly being used by consumers, clinicians, and policy-makers as part of their decision-making process [3]. Although these reviews are highly regarded, their frequency of publication and factors associated with their publication remains unknown. If factors such as funding are associated with subsequent publication this may imply that Cochrane reviews are also subject to publication bias.

Publication bias occurs when “investigators, reviewers, and editors submit or accept manuscripts for publication based on the direction or strength of the study findings”[4]. Publication bias also occurs when studies with certain characteristics (e.g., favourable results, funding from organizations with vested interests, such as pharmaceutical companies or the tobacco industry) are published quicker than those without these characteristics [5]. Publication bias has been extensively examined for individual studies (e.g., randomized trials) [4], [6]–[11], but is under-explored for systematic reviews [12]–[14].

Cochrane reviews can be followed over time to examine whether certain factors are associated with their publication. The process for publishing a Cochrane review includes the following: 1) title (or topic) registration to ensure that the review is unique to The Cochrane Library, 2) publication of a protocol, which outlines the review plan, 3) conduct of the review, 4) publication of the review report, and 5) update of the review, which usually occurs every two years [1]. Cochrane reviews can be published elsewhere, yet they should be published in The Cochrane Library first. All Cochrane protocols and their respective reviews are provided with a unique Cochrane identification number, which allows both to be followed over time. We conducted a retrospective cohort study to examine the frequency of published and unpublished Cochrane reviews and determine the protocol factors that predict the publication of Cochrane reviews.

## Methods

### Cohort sample acquisition

A new issue of The Cochrane Library is published quarterly along with a CD with all of its contents. We obtained all Cochrane Library CDs from inception from the UK Cochrane Centre and Canadian Cochrane Network and Centre. In order to allow time for publication, we selected all new protocols from 2000 (Issues 2 to 4) and 2001 (Issue 1) [4], [8]–[11]. The CD indicates when a Cochrane protocol is new to that particular issue.

The unique Cochrane identification number was entered into Issue 1, 2008 of The Cochrane Library to determine the publication status. Authors of Cochrane protocols that could no longer be found in The Cochrane Library were contacted for further information. When a response from the authors was not received, the CRG coordinator responsible for the Cochrane protocol was contacted.

The new protocols arising from the Cochrane CDs were subsequently screened to ensure that they were eligible for the study. Cochrane protocols that were split into more than one Cochrane review, taken over by another review group, published in the same issue as the corresponding Cochrane review itself, published later than the review publication or published prior to Issue 2, 2000 were excluded.

### Data abstraction

A 37-item data abstraction form was developed by two investigators (ACT, DM) and pilot-tested. Descriptive characteristics (country of conduct, population examined, number of authors, number of protocols [multiple protocols indicating that changes to the original review plan occurred], number of unique Cochrane identification numbers [some of the reviews had multiple numbers]), planned methodology (observational versus experimental study inclusion, number of databases searched, number of primary outcomes, inclusion of unpublished material, language inclusion, assessment of publication bias, assessment of heterogeneity), and other characteristics (gender of corresponding author and whether they were a healthcare provider, number of updates, funding) were abstracted from the Cochrane protocols by one investigator (ACT). Data were also abstracted from the original version of the Cochrane review, such as the timing of publication and whether it was subsequently updated. Random data checks were made by two investigators, independently (ACT, MHC).

Two time points were abstracted for the analysis from all included protocols and their subsequent reviews. The first was the *version first published online* date of publication from The Cochrane Library citation and the second was the *most recent substantive amendment date* from the cover page of the Cochrane protocol and associated completed review. As Cochrane reviews are published quarterly, the *version first published online* date is truncated to four time points per year. As such, it was decided that the *most recent substantive amendment date* would be used for the primary analyses while the *version first published online date* would be used for sensitivity analyses. The most recent substantive amendment date always occurs prior to the publication date, resulting in more than eight years of follow-up data.

### Data analysis

Time-to-publication analyses were conducted using the Kaplan-Meier method, which is often used to estimate time-related events and takes into account censored data (i.e., losses to the sample that occur prior to the final outcome) [15]. Cochrane reviews that remained unpublished at the time of study were censored on January 23, 2008 (i.e., the publication date of Cochrane Library, Issue 1, 2008). Cox proportional hazards models (regression models often used to examine time-dependent factors) were then used to predict the time to publication of Cochrane reviews. Hazard ratios and 95% confidence intervals were calculated. The hazard ratio is the effect of an explanatory variable on the hazard or risk of an event and can be thought of as an estimate of the relative risk (i.e., the risk of an event, in this case the risk of being unpublished, relative to exposure, such as, lack of funding, negative results). Variables chosen for the univariate and multivariate analyses were based on *a priori* consideration of most plausible predictors for time to publication. Both univariate and multivariate models and interactions between variables were assessed. Statistical analyses were conducted with SAS, version 9.0 (SAS Institute, Cary, North Carolina). This analysis is consistent with research on the publication status of individual studies (e.g., randomized trials) [4], [8]–[11], providing the opportunity to compare our results with these studies.

## Results

### Frequency of published Cochrane reviews

**Figure 1:**
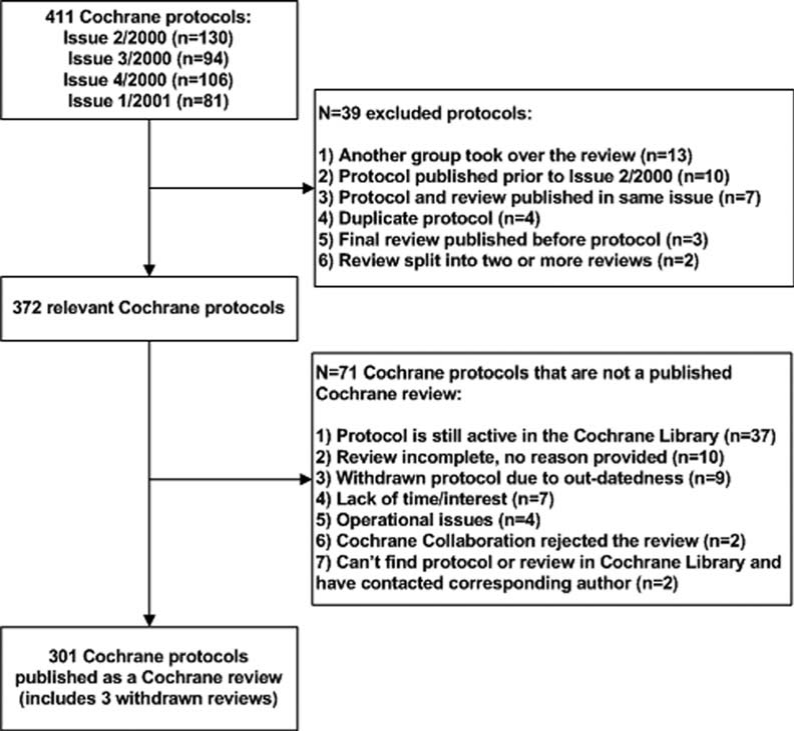
Study flow. The cohort included a total of 411 Cochrane protocols and 379 (90.5%) remained after excluding 39. Of these protocols, 19.1% (71/372) were never published as full Cochrane reviews while 80.9% (301/372) were published in full.

There were a total of 411 Cochrane protocols published in Issues 2 to 4, 2000 and Issue 1, 2001 of The Cochrane library. After excluding 39 protocols 372 (90.5%) remained in our sample (Figure 1). Of these protocols, 19.1% (71/372) were not published as full Cochrane reviews at the time of this study while 80.9% (301/372) were published in full. Only 33.2% (100/372) of the reviews were subsequently updated.

Reasons for non-publication as final reviews included that the protocol is still active in The Cochrane Library and a corresponding review has never been conducted (52.1%, 37/71), the review authors acknowledged that the review is incomplete but no reason was provided (14.1%, 10/71), the protocol was withdrawn due to out-datedness (12.7%, 9/71), the Cochrane review authors lacked time or interest (9.9%, 7/71), the reviewers experienced operational issues (e.g., the lead author changed jobs; 5.6%, 4/71), and the Cochrane Collaboration rejected the review (2.8%, 2/71). Information about two protocols (2.8%) was not provided after contacting the corresponding author of the review.

We contacted the corresponding author or CRG coordinator for the 71 reviews that were unpublished as of January 2008 to determine the stage that the review was at, as well as to inquire whether the review was ever published elsewhere. Sixty-eight responses (96%) were received. The review was incomplete (stage not reported, 52.9%, 36/68), complete but never published in Cochrane (10.3%, 7/68), a draft manuscript was compiled (8.8%, 6/68), at the literature search stage (7.4%, 5/68), in peer review (7.4%, 5/68), at the analysis stage (5.8%, 4/68), and at the data abstraction stage (7.4%, 5/68). Only 13.2% (9/68) of the reviews were published elsewhere, one of which was published as a book chapter.

### Cochrane protocol characteristics

The majority of the corresponding authors were based in the United Kingdom (39.5%, 147/372), while 13.4% (50/372) were based in Australia, 7.3% (27/372) in Canada, and 7.0% in the United States (26/373; Table 1). The median number of authors per protocol was 3 (range: 1–22). Almost 3% (10/372) of the reviews had two published protocols. Approximately 7% (27/372) of the protocols had two unique Cochrane identification numbers, possibly indicating inconsistent editorial practices.

**Table 1:** Cochrane review characteristics.

Item	Total: 372 Cochrane reviews
**Descriptive characteristics**	
*Country of conduct: n (%)*	
United Kingdom	147 (39.5)
Australia and New Zealand	50 (13.4)
Canada	27 (7.3)
United States of America	26 (7.0)
Italy	14 (3.8)
Netherlands	12 (3.2)
Brazil	9 (2.4)
France	8 (2.2)
China	7 (1.9)
Denmark	7 (1.9)
South Africa	7 (1.9)
Spain	7 (1.9)
Other	38 (10.2)
Not reported	13 (3.4)
*Population examined: n (%)*	
Neonates only	21 (5.6)
Children only	11 (3.0)
Adolescents only	1 (0.3)
Adults only	61 (16.3)
Women only	49 (13.2)
Men only	4 (1.1)
Elderly only	4 (1.1)
Children and adolescents	13 (3.5)
Children, adolescents and adults	2 (0.5)
Adolescents and adults	5 (1.3)
Adolescents, adults and elderly	1 (0.3)
All	200 (53.8)
*Number of authors: median (range)*	3 (1, 22)
*Review had two protocols: n (%)*	10 (2.7)
*Review had two unique Cochrane identification numbers: n (%)*	27 (7.3)
**Methodological characteristics**	
*Type of reports to be included in the reviews: n (%)*	
Observational only	0 (0)
Experimental and quasi-experimental only	358 (96.2)
Both	14 (3.8)
*Number of databases to be searched: median (range)*	4 (1, 22)
*A primary outcome was reported: n (%)*	274 (73.7)
*Number of primary outcomes: median (range)*	1 (1, 20)
Reviews with multiple primary outcomes: n (%)*	135 (49.3)
*Language inclusion: n (%)*	
English only	6 (1.6)
Mixed languages only	5 (1.4)
All languages	128 (34.4)
Not reported	233 (62.6)
*Publication bias was to be assessed: n (%)*	75 (20.2)
*Heterogeneity was to be assessed: n (%)*	282 (75.8)
**Other characteristics**	
*Gender of corresponding author: n (%)*	
Female	132 (35.5)
Male	192 (51.6)
Unclear	48 (12.9)
*Corresponding author was a healthcare provider: n (%)*	76 (20.4)
*Number of reviews with funding: n (%)*	216 (58.1)
*Type of funding source: n (%)‡*	
Government only	59 (27.3)
Not-for-profit organization only	100 (46.3)
Insurance company only	1 (0.5)
Government and not-for-profit organization	51 (23.6)
For-profit organization and government	2 (0.9)
For-profit and government and not-for-profit	3 (1.4)

**Notes:** ^*^ Denominator is number of reviews with a primary outcome (n = 274), ^†^ denominator is published reviews (n = 301), ^‡^ denominator is number of reviews with funding (n = 216).

The majority of the protocols indicated a plan to include experimental (e.g., randomized controlled trials) and quasi-experimental (e.g., interrupted time series) primary studies (96.2%, 358/372; Table 1). Almost 75% of the protocols reported a planned primary outcome (73.7%, 274/372) and the median number of planned primary outcomes per protocol was 1 (range: 1–22). When reported, the majority of the protocols planned to include all languages (34.4%, 128/372) and assess for heterogeneity (75.8%, 282/372), yet only 20.2% (75/372) planned to assess for publication bias.

A little over half of the protocols reported a funding source (58.1%, 216/372; Table 1). This was predominantly a not-for-profit funder (46.3%, 100/216); while 27.3% (59/216) reported funding from a government agency and 23.6% (51/216) reported joint government and not-for-profit funding. Few protocols reported for-profit organization funding, which is a Cochrane mandate [1] and few of the corresponding authors reported being a healthcare provider (20.4%, 76/372).

### Survival analysis

**Figure 2:**
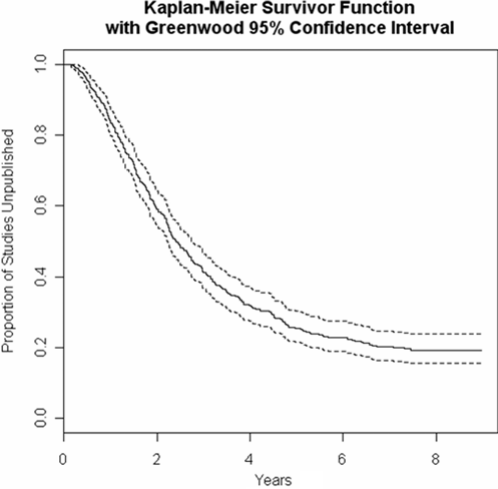
Kaplan-Meier Curve for the time to publication of Cochrane reviews and 95% confidence intervals. The Kaplan-Meier Curve displays that the proportion of unpublished Cochrane reviews decreases over time.

The median time to publication using the *most recent substantive amendment date* was 2.4 years (range: 0.15 to 8.96 years; interquartile range, IQR: 3.8 years; Figure 2). This was similar to the sensitivity analysis (i.e., the *version first published online date*), which was 2.24 years (range: 0.25 to 7.75 years; IQR: 3.7 years). Of the variables chosen for the univariate analyses, four were significant and entered into the multivariate analyses: having two protocols (p = 0.001); an updated review (p<0.0001), number of authors (p = 0.008); and number of primary outcomes (p = 0.002). There was also a trend towards significance for the language inclusion variable (p = 0.06). These five factors were subsequently used in the Cox proportional hazard model (Table 2).

**Table 2:** Factors predicting the time to publication of Cochrane reviews

Factor	Univariate Hazard Ratio* (95% CI)	p-value	Multivariate Hazard Ratio* (95% CI)	p-value
Language inclusion (including all vs. including mixed languages and not reported)	1.27 (1.00, 1.61)	0.04	1.31 (0.69, 2.50)	0.42
Language inclusion (not reported vs. reported)	0.78 (0.62, 0.98)	0.03	1.00 (0.52, 1.91)	0.10
Review has two published protocols vs. one published protocol	0.27 (0.10, 0.72)	0.01	0.33 (0.12, 0.90)	0.03
Number of primary outcomes reported in the protocol	1.05 (0.77, 1.44)	0.00	1.02 (0.98, 1.05)	0.23
Number of authors on the protocol	0.90 (0.84, 0.98)	0.01	0.94 (0.86, 1.03)	0.17
Review subsequently being updated	1.87 (1.47, 2.35)	<0.001	1.78 (1.39, 2.33)	<0.0001

**Note:** ^*^ Hazard ratios indicate the relative hazard to the time to publication. Numbers above 1 indicate an decreased time to publication, numbers below 1 indicate an increased time to publication.

**Abbreviation:** CI confidence interval.

In the multivariate analyses only two of the variables were significant. A shorter time to publication was associated with the review being an update (hazard ratio, HR 1.80 [95% CI: 1.39, 2.33)] and a longer time to publication was associated with the review having two published protocols (HR 0.33 [95% CI: 0.12, 0.90]; Table 2). Sensitivity analysis based on the *version first published online* date produced similar results.

## Discussion

We conducted a retrospective cohort study of Cochrane protocols to provide data on the average time to publication of Cochrane reviews and factors associated with their publication. Our results indicate that for every four published Cochrane reviews, one review remained unpublished based on one year of Cochrane protocols. As Cochrane reviews are regarded as being scientifically rigorous, this finding is disquieting. As a major contributor to the systematic review literature, we believe that all Cochrane protocols should be completed and published as Cochrane reviews. For the unpublished Cochrane reviews, only a minority (13.2%) were published elsewhere, indicating a major loss of information being publicly available, as well as wasted scarce resources.

A little more than half (52.1%) of the unpublished reviews were still active Cochrane protocols in The Cochrane Library. This indicates a lack of consistency in the Cochrane Collaboration's editorial procedures, as some of the protocols were withdrawn due to out-datedness while others were not. Another editorial inconsistency was the finding that 7% of the included protocols had two unique identification numbers. The Cochrane Library is unusual in that there is no single person directly responsible for its quality assurance. We hope that with the appointment of the Library's new editor-in-chief, the number of unpublished Cochrane reviews will decrease substantially.

Our results indicated that the median time to publication of the completed Cochrane review from the published protocol was 2.4 years, and some reviews took as long as 9 years to be published (using the *most recent substantive amendment date*). Our results are consistent with another study that examined the time to publication from submission to final publication of the review [14]. However, our time frame is double that reported elsewhere [15], as this study examined a different time period than this study did [15].

In this study, a longer time to publication was associated with the review having two protocols. Strategies to decrease time to publication should be considered. These may include providing support to reviewers when protocol changes occur and streamlining the publication process to decrease the time to publication of Cochrane reviews [16].

As noted elsewhere, updating systematic reviews is of paramount importance because some health care interventions currently known to be effective may be shown to be ineffective or harmful in the future and new interventions or health outcomes may emerge [17], [18]. Our results indicate a shorter time to publication associated with the review subsequently being updated. This could be due to a variety of reasons, such as a quickly evolving clinical content area or a highly motivated Cochrane review team. A recent study examined indicators predicting when systematic reviews go out of date [15]. These analyses found that shorter time to update was associated with the cardiovascular content area (i.e., indicating a quickly evolving clinical area) and heterogeneity being present or suspected in the review (i.e., indicating a motivation to examine unstable results).

The current Cochrane guidance is to update their reviews every 2 years [1]. Although our cohort spans over 8 years, only a third of the reviews were updated and only 2 out of the entire sample had 3 updates. For Cochrane reviews (as any other systematic reviews) to maintain their currency, a more active policy should be considered to ensure that a much higher proportion is kept up-to-date. This could include international harmonization of aspects of the updating process and having other authors finish the update when too much time has elapsed.

The reasons for unpublished Cochrane reviews seem to be different than the reasons for unpublished individual studies (e.g., trials). For clinical trials, there is a trend towards shorter time to publication when they are sponsored by private industry (e.g., pharmaceutical companies) [9], [11] and a higher likelihood of publication when they are funded [19]. Our findings are consistent with a recent survey on the publication practices of systematic reviewers. In this survey, the most commonly reported reasons for not publishing Cochrane reviews included lack of time, the manuscript being rejected, and operational issues (Andrea Tricco personal communication). Members of the investigative team are currently involved with research exploring these issues.

This study has some limitations. Only one investigator abstracted all of the data, which could have led to inaccuracies. Furthermore, we did not examine all of the review factors associated with the time to publication and the reasons for publishing Cochrane reviews elsewhere often were not provided by the review authors. However, our cohort includes one year of data with a large number of Cochrane protocols, a high response rate was attained for the 71 unpublished reviews, and two investigators performed random data checks and resolved any issues with the data. Furthermore, the Cochrane review factors associated with the time to publication have been examined elsewhere recently (Andrea Tricco, personal communication).

In conclusion, only about 80% of Cochrane protocols were published as full reviews after more than 8 years of follow-up. The median time to publication was nearly two and a half years and some reviews took considerably longer. We recommend that the Cochrane Collaboration have consistent editorial policies, streamline the review process to decrease the time to publication, provide support for review authors when changes to the protocol occur, and provide a better infrastructure for updating Cochrane reviews.
